# Hepatitis B Vaccination Status in Chronic Kidney Disease: Experience at Pakistan Institute of Medical Sciences

**DOI:** 10.7759/cureus.5282

**Published:** 2019-07-30

**Authors:** Amna Amjad, Jais Kumar, Noureen Chaudary, Kishore Kumar, Chaudhary Muhammad Junaid Nazar, Khushal Khan

**Affiliations:** 1 Nephrology, Pakistan Institute of Medical Sciences, Islamabad, PAK; 2 Medicine, Pakistan Institute of Medical Sciences, Islamabad, PAK; 3 Nephrology and Renal Transplantation, Maroof International Hospital, Islamabad, PAK; 4 Gastroenterology, Shifa International Hospital, Islamabad, PAK

**Keywords:** chronic kidney disease, hepatitis b, vaccination, chronic kidney disese, hepatitis b virus, chronic kidney diseas, chronic kidney disease heaptitis b vaccination

## Abstract

Objective

To know the status of vaccination against hepatitis B in chronic kidney disease patients, and to see the association of vaccination status with various factors.

Materials and Methods

A cross-sectional observational study was conducted in the Department of Nephrology, Pakistan Institute of Medical Sciences, Islamabad, from December 2015 to May 2016. Patients reporting in the outpatient department or admitted with the diagnosis of chronic kidney disease, whether on dialysis or not, were included in the study. Patient’s information like the status of hepatitis B vaccination, age, gender, socioeconomic status, education and duration of chronic kidney disease were collected on a specially designed proforma.

Results

A total of 451 patients were included in the study, 57.43% were male and 42.57% were female. Mean age was 43.76±17.12 years. About 69% of the patients were from low socioeconomic class, 31% from the middle or higher middle class. Most of the patients were either uneducated (32.59%) or only had eight years of school education (33.04%). Only 19.9% of patients were vaccinated against hepatitis B virus.

Conclusion

Status of vaccination against hepatitis B virus in chronic kidney disease patients is not satisfactory. It is strongly associated with socioeconomic class.

## Introduction

Hepatitis B virus (HBV) infection is a serious global health problem. It is an important cause of cirrhosis of the liver and hepatocellular carcinoma. It is estimated that it affects 350-400 million people worldwide [1]. The prevalence of HBV infection is not same globally; it is highest in the WHO Western Pacific Region and the WHO African Region, where > 6% of the adult population is infected. Prevalence is 3.3% in the Eastern Mediterranean Region, about 2% in the WHO South-East Asia Region and 1.6% in the WHO European Region in the general population [1]. Only 0.7% of the population of the WHO Region of the Americas is infected [1]. The prevalence in Pakistan is about 4% [2]. Prevalence is very high worldwide among some high-risk groups; like in drug abusers, it is about 30% whereas in HIV patients it is 15% [3,4].

Chronic Kidney Disease (CKD) patients, especially those requiring hemodialysis, are among high-risk groups[5]. Increased risk of acquiring HBV infection in these patients is due to shared hemodialysis equipment, increased exposure to blood products, frequent breaching of skin, compromised immune system and high prevalence rates of hepatitis B infection among hemodialysis population[6]. HBV infection in CKD patients varies and correlates with the prevalence in the general population. It is generally decreasing especially in the developed world; in the US it fell from 7.8% in 1976 to 1% in 2002[5]. The epidemiology of HBV infection among CKD and dialysis patients in the less developed world is not well known. There are scattered reports, typically single-center surveys; with the rate of chronic HBsAg carriers ranging between 2% and 20% [6].

Preventive measures are important to reduce the prevalence of hepatitis B infection in the general population as well as in high-risk groups like CKD patients. Hepatitis B virus can remain viable on medical supplies, utensils and other objects for one week on room temperature [7]. Thus vaccination against hepatitis B is the most effective way to prevent it. The complete vaccination is effective in 95% of infants, children and young adults [8]. Although the response is not good in chronic renal failure patients, up to 40% were found non-responder in some studies still, routine vaccinations of patients, healthcare workers and use of erythropoietin instead of blood transfusions has dramatically reduced the prevalence of HBV infection in hemodialysis patients [9,10]. Hepatitis B infection was found 70% lower among vaccinated patients as compared to non vaccinated hemodialysis patients [11].

Hepatitis B vaccination is recommended for all chronic kidney disease patients. Higher dosage and increased number of doses (four doses) are advised for these patients because their response is not as good as in general population[12]. It is advised that these patients should be vaccinated at an earlier stage and before the start of hemodialysis because the response is good with higher eGFR [13]. Despite strong recommendations, this is not practiced frequently even in the developed world. Only 46% of dialysis units were routinely immunizing patients according to the Renal Association's recommendations in the United Kingdom as shown by Ray et al. [14].

No data is available regarding the Hepatitis B vaccination status in CKD patients in Pakistan; this study aimed to see the status of hepatitis B vaccination among CKD patients and the factors associated with it. 

## Materials and methods

This cross-sectional, observational study was conducted at the nephrology department of Pakistan Institute of Medical Sciences, Islamabad, from December 2015 to May 2016. Patients reporting to the nephrology outpatient department (OPD) or admitted in nephrology ward with the diagnosis of CKD whether on dialysis or not, age > 15 years, were included in the study. CKD was defined as "decreased kidney function shown by glomerular filtration rate (GFR) of less than 60 mL/min per 1·73 m^2^, or markers of kidney damage, or both, of at least three months duration, regardless of underlying cause" [15]. The patients tested positive for Hepatitis B surface antigen and newly diagnosed CKD patients were excluded. Patients were included consecutively after obtaining informed consent from the patient or his close relative wherever relevant. Approval in this regard was taken from the Hospital Ethical Committee. Patient’s information like the status of hepatitis B vaccination, age, gender, socioeconomic status, education and duration of CKD were collected on a specially designed proforma. 

Data was entered on SPSS version 17. Quantitative analysis like age and duration of CKD was measured ±SD. Frequencies were calculated for gender, education, socioeconomic status and vaccination status of Hepatitis B. Effective modifiers like age, duration of CKD, education and socioeconomic status were controlled by stratification. Chi-square test was applied to see the relationship between different factors and status of vaccination. P-value ≤ 0.05 was considered as significant.

## Results

Total 451 patients were included in the study, 57.43% (n=259) were male and 42.57% (n=192) were female. Mean age and duration of CKD were 43.76±17.12 years and 9.94±2.78 months respectively. Most of the patients were from poor socioeconomic class (n=311; 69%), about 31% (n=140) were from the middle or higher middle class. Majority of the patients were either uneducated (n=147; 32.59%) or only had eight years of school education (n=149; 33.04%). Only 19.9% (n=90) patients were vaccinated against hepatitis B virus. There was no significant association between hepatitis B vaccination and age, gender, education or duration of CKD of the patients (Tables 1, 2). The status of hepatitis B vaccination was significantly associated with the socioeconomic class (Table 3). 

**Table 1:** Status of Hepatitis B vaccination in CKD patients with respect to age groups The total sample size was of 451 patients. These patients were divided according to age. Out of these 37 were less than 20 years of age. 28 (75.7%) of these patients were not vaccinated, whereas, only 9(24.3%) were vaccinated for hepatitis B. 83 were between the age of 21 to 30. Amongst these 64(77.1%) patients were not vaccinated and 19(22.9%) were vaccinated. 75 patients were between the age of 31-40 with only 17(22.7%) vaccinated and 58(77.3%) were not vaccinated. 188 patients were between the ages of 41-60. 150(79.7%) out of these were not vaccinated whilst 38(20.2%) were vaccinated. 68 patients were over the age of 60 with only 7(10.3%) patients vaccinated and 61(89.7%) were unvaccinated.

Age Groups (Years)	HEPATITIS B VACCINATION		Total
Yes n=90	No n=361
≤ 20	9(24.3%)	28(75.7%)	37
21 to 30	19(22.9%)	64(77.1%)	83
31 to 40	17(22.7%)	58(77.3%)	75
41 to 50	21(18.8%)	91(81.3%)	112
51 to 60	17(22.4%)	59(77.6%)	76
>60	7(10.3%)	61(89.7%)	68
p=0.348			

**Table 2:** Hepatitis B vaccination status in CKD patients with respect to gender and the duration of CKD When the difference of treatment between genders was compared, it was seen that 153(79.7%) of the male patients had not been vaccinated and 39(20.3%) had been vaccinated. While 208(80.3%) of the female patients had not been vaccinated and 51(19.7%) had been vaccinated. 311 patients had CKD for 6-10 months. Out of these 253(81.4%) had not been vaccinated and  58(18.6%) had been vaccinated. 140 patients had CKD for over 10 months. 108(77.1%) of these had not been vaccinated and 32(22.9%) had been vaccinated.

Variables		Hepatitis B vaccination status		p value
Yes n=90	No n=361
Gender	Male	39(20.3%)	153(79.7%)	p=0.87
	Female	51(19.7%)	208(80.3%)	
Duration of CKD	6 to 10 months	58(18.6%)	253(81.4%)	p=0.30

**Table 3:** Hepatitis B vaccination status in CKD patients with respect to socioeconomic status Majority of the patients belonged to poor socioeconomic class 69% (n=311), out of these 266(85.6%) were unvaccinated and  45(14.4%) were vaccinated. Whereas, about 31% (n=140) were from the middle or higher middle class. 95 (67.8%) of these patients were unvaccinated and only 45(32.1%) patients were vaccinated. A large number of the patients were either uneducated (n=147; 32.59%) or only had eight years of school education (n=149; 33.04%).

SOCIOECONOMIC STATUS	HEPATITIS B VACCINATION		Total	
Yes n=90	No n=361	p Value
Poor Class	45(14.4%)	266(85.6%)	311	
Middle Class	43(31.4%)	94(68.6%)	137	p=0.038
Upper Middle Class	2(66.7%)	1(33.3%)	3	

## Discussion

Our study shows that only 20% of patients with CKD were vaccinated against hepatitis B virus despite the strong recommendations of nephrology societies and the Centre for Disease Control and Prevention. The status of vaccination is significantly associated with the socioeconomic class in our study, but it is not associated with age, gender or duration of CKD.

Vaccination status of CKD patients against hepatitis B virus is not well studied, though many studies are available regarding the response of hepatitis B virus vaccine in CKD patients. A study conducted in the United Kingdom in the early 90s showed that only 5% of dialysis units were routinely immunizing the patients [16]. The number of vaccinated patients is increasing in the USA, the percentage increased from 47% to 56% during 1997-2002 [17]. Our results showed that only 20% of patients were vaccinated against HBV. We could not find any local data to compare our results. But data from other developing countries shows that vaccination status is much better as compared to our results. Almost 60% of patients of CKD were found vaccinated against HBV in Brazil, another 15% were incompletely vaccinated [18]. Our results are alarming as CKD patients are among high-risk groups and vaccination is the most effective way to prevent HBV infection along with segregation of Hepatitis B patients and their equipment from other CKD patients and general infection control measures [19]. This low rate of vaccination indicates that nephrologists and dialysis centers are not following the recommendations most of the time.

The United States Renal Data System 2011 Annual Data Report showed that initiation of hemodialysis in 2009 was much higher in males as compared to females [20]. Maric also showed that men have an increased risk of developing CKD in diabetes [21]. Our results are comparable, 57.4% of our patients were male. But there was no association between the gender and vaccination against hepatitis B virus in CKD patients. Most of our CKD patients were above 40 years of age; this is in accordance with other studies. A study conducted in India showed a mean age of 51 years in CKD patients; in a Chinese study, it was 63.6 years[22,23]. Again we did not find any significant association between the age of patient and duration of CKD with the vaccination status. Studies have shown that older age was associated with a lower vaccination rate among the general population, but we could not found any data regarding age and hepatitis B vaccination status in CKD patients to compare our results [24,25].

Most of our CKD patients were from lower socioeconomic class, same was concluded by Hossain et al. in a study from UK [25]. Socioeconomic status of the patients was significantly associated with HBV vaccination rate in our study; vaccination rate was lower among patients from lower socioeconomic class. A study conducted by Ertekin et al. also showed similar results in the general population[26]. This may be due to non-affordability and lack of awareness about the hepatitis B virus and the significance of vaccination against hepatitis B virus, in patients from the lower and lower-middle class.

Our study has some limitations: only one nephrology unit having one dialysis center was included in the study and only a few factors were studied related to the vaccination status. We recommend studies in future including more centers, and more factors need to be studied influencing the vaccination status.

## Conclusions

Vaccination status of the chronic kidney disease patients against hepatitis B virus infection is very low despite strong recommendations of the nephrology societies and Center for Disease Control and Prevention. There is a significant association between vaccination status and socioeconomic class. Some special measures should be taken to increase the vaccination rate, especially in patients from lower socioeconomic class.
